# Experiences of insomnia among older people living in nursing homes A qualitative study

**DOI:** 10.1080/17482631.2025.2476788

**Published:** 2025-03-19

**Authors:** Eva Hjort Telhede

**Affiliations:** School of Health and Welfare, Halmstad University, Halmstad, Sweden

**Keywords:** Nursing homes, older people, qualitative study, sleep disturbances, nsomnia

## Abstract

**Purpose:**

The study aimed to explore older people’s experiences of insomnia in nursing homes.

**Method:**

This qualitative study used an inductive approach with semi-structured interviews involving 19 older people (aged 67–101 years) from nine nursing homes in southwestern Sweden. The older people were purposively selected based on insomnia criteria according to the International Classification of Diseases (ICD-10, G47.0) and cognitive competence according to the Standardized Mini-Mental State Examination (S-MMSE). The interviews were analysed using qualitative content analysis.

**Results:**

Two categories were identified: Valuing good sleep and Disruptive influence on sleep, with the subcategories of internal and external disturbances. Older people expressed that sleep was crucial to their well-being, and poor sleep quality negatively influenced their mood and physical health. They experienced internal disturbances, such as anxiety and physical discomfort, as causing sleep disturbances, but also external aspects that included environmental disturbances, reduced activity levels, loneliness, and worry about which nursing staff was on duty.

**Conclusion:**

This study highlights the critical role of sleep in maintaining energy, coping with daily life, and ensuring overall well-being for older people in nursing homes.

## Introduction

The ageing population is rapidly increasing, creating both challenges and opportunities for healthcare systems. As the number of older people, typically defined as those aged 65 and older, rises, there will be an increasing need for healthcare services, particularly for addressing chronic conditions such as cardiovascular disease and dementia (National Council on Aging, 2023). Older people, characterized by varying levels of independence, often experience multimorbidity, which is defined as the coexistence of multiple chronic conditions or illnesses along with age-related disabilities.

Among the most common health problems in this group is insomnia disorder, a condition with profound health implications that extends beyond night-time symptoms. According to the ICD-10, insomnia is defined as persistent difficulty falling asleep, staying asleep, or waking up too early, despite having adequate opportunities for sleep (American Academy of Sleep Medicine, 2014; Riemann et al., 2020; World Health Organization [WHO], 2019). Particularly in nursing homes, insomnia is not only highly prevalent but also exacerbated by institutional factors such as rigid care routines, environmental disturbances, and limited opportunities for physical activity during the day (Erdogmus et al., 2022). Research suggests that sleep disturbances such as insomnia are more pronounced in institutionalized settings than among older people living in the community, with prevalence rates ranging from 50% to 70% in nursing homes compared to 30% to 50% among community-dwelling older adults (Fang et al., 2019; Smagula et al., 2016).

Age-related physiological changes, including reduced melatonin production and alterations in circadian rhythms, further exacerbate insomnia disorder in this population (Patel et al., 2018; Irwin, 2019). Additionally, multimorbidity—where multiple chronic conditions coexist—makes insomnia more prevalent among older people, contributing to its persistence (Harrison et al., 2020; Sivertsen et al., 2015). About 40% to 70% of older people experience sleep disturbances, including insomnia, which can lead to fragmented sleep and significantly affect both physical and mental health (Harrison et al., 2020).

The influence of insomnia extends beyond night-time symptoms, influencing daytime functioning, cognitive performance, and mood. It has been linked to an increased risk of cognitive decline, mood disorders such as depression and anxiety, and a reduced quality of life (Hirshkowitz et al., 2015; Lee et al., 2020). Despite recommendations that older people should achieve 7–8 hours of sleep per night, many nursing homes face sleep deprivation due to environmental and structural constraints (National Sleep Foundation, 2015). Factors such as nursing staff routines, night-time monitoring, medication schedules, and limited autonomy over sleep schedules exacerbate insomnia disorder, making it difficult for older people to achieve adequate rest and sleep (Gupta et al., 2022, Li et al., 2021; Pottie et al., 2018).

Beyond these physiological influences, psychosocial factors further influence sleep quality. Social isolation, which is common among older people in nursing homes, has been associated with heightened feelings of anxiety and depression, which in turn disrupt sleep patterns, as in insomnia (Sexton et al., 2020). Additionally, the lack of meaningful social interactions and engagement in daily activities often leads to daytime sleepiness and poor night-time sleep, and in some cases, people with depression may sleep excessively, spending much of their days in bed (Gupta et al., 2022; Zhang et al., 2022). Physical discomfort due to chronic pain or illness contributes to difficulty falling asleep and maintaining sleep, which can lead to persistent sleep disturbances that meet the diagnostic criteria for insomnia, such as difficulty initiating or maintaining sleep despite having adequate sleep opportunities (Li et al., 2021; Sivertsen et al., 2015). Despite the well-documented prevalence of insomnia in older people, there is a critical gap in research regarding how older people in nursing homes perceive and experience their sleep difficulties. Understanding their perceptions of the elements influencing sleep and how insomnia influences older people’s daily lives is crucial. Exploring the experiences of older people in nursing homes provides insights into the multifaceted nature of insomnia from their perspective. Previous studies emphasize the importance of integrating subjective experiences in order to understand the underlying causes of insomnia and to develop tailored interventions to improve sleep and overall well-being (Blytt et al., 2020; Dörner et al., 2023; Hjort Telhede et al., 2022; Tranah et al., 2018). Blytt et al. (2020) highlight the need for individualized care plans based on qualitative assessments of insomnia among nursing homes. Similarly, Dörner et al. (2023) emphasis the importance of care based on the individual challenges older people face regarding sleep.

Although some research has explored older people’s subjective sleep experiences in nursing homes, the available studies remain limited. Li et al. (2021) and Lichstein et al. (2009) examined self-reported sleep disturbances among community-dwelling older people, identifying fragmented sleep, frequent awakenings, and high rates of insomnia, particularly among women and individuals with lower educational levels. These studies highlight the widespread nature of sleep disturbances and their consequence on well-being.

Similarly, O’Keeffe and Lavan (2019) investigated sleep difficulties among older people in nursing homes, emphasizing the role of environmental noise, physical discomfort, and health-related factors. Gürsoy and Silay (2024) further supposed that poor sleep quality and daytime fatigue are prevalent in this population, negatively affecting the quality of life. They support integrating insomnia management into nursing home care. More recently, Gordon et al. (2024) explored registered nurses’ (RNs) perspectives on insomnia in aged care, revealing that while RNs acknowledge the consequences of sleep disturbances, their ability to intervene is hindered by existing care practices and limited training.

While prior research has established that sleep problems are common among older people and influenced by multiple factors, the perspectives of nursing older people in nursing homes remain overlooked. Most existing studies focus on clinical assessments rather than the lived experiences of older people in these settings. This study aims to address this gap by exploring the subjective insomnia experiences of older people in nursing homes.

### Aim

The study aimed to explore older people’s experiences of insomnia in nursing homes.

## Materials and methods

This study employed a descriptive qualitative design, utilizing semi-structured interviews with older people in nursing homes followed by an inductive content analysis (Elo & Kyngäs, 2008; Graneheim & Lundman, 2004). The data presented here were drawn from a larger research project, with previous findings published in Telhede et al. (2024). These data were collected during the same research phase but have not been analysed until now. The current study conducted a new analysis of the collected data to explore older people’s experiences of sleep problems such as insomnia in nursing homes. While the participants in both studies were the same, the material presented in this study is distinct and has not been previously analysed or published. A qualitative approach was chosen to capture the personal and subjective experiences of insomnia, providing insight into the emotional, psychological and social aspects that are essential to understanding the full impact of the condition on older people’s lives

### Sample

Data were collected from 13 nursing homes in five municipalities in southwestern Sweden. A purposive sampling strategy was employed, where nursing home managers were informed about the study’s purpose and assisted in selecting participants. The study involved 19 older people (4 men and 15 women, ranging in age from 67 to 101 years) who met the criteria for insomnia and the sociodemographic characteristics described in Table 1.

**Table 1.** Sociodemographic data of the informants (*n* = 19).

Category	Female	Male
Sex	15 (79%)	4 (21%)
Age (years)	86 (77–98)	82 (67–101)
Nursing homes	Urban areas 3 (33%)	Rural areas 6 (67%)

Values are given as n (%) or mean (range).

The participants did not have a formal diagnosis of insomnia, but they were selected based on symptoms corresponding to the insomnia criteria. Insomnia was defined according to the Swedish version of the International Classification of Diseases (ICD-10, G47.0), which includes difficulties in falling asleep, staying asleep, or obtaining adequate sleep. Insomnia is considered chronic when it occurs at least three times per week for a minimum of one month and causes significant distress or interferes with daily activities (American Psychiatric Association, 2013).

Cognitive competence was assessed using the Standardized Mini-Mental State Examination (S-MMSE), with participants needing to score above 20 points to ensure they were capable of providing independent responses (Folstein et al., 1975; Molloy & Standish, 1997; Palmqvist et al., 2011). The sample size was determined based on the principle of data saturation. This study reached saturation after interviewing 19 participants, as no new data emerged afterwards.

## Data collection

Data collection took place through interviews conducted between March 2020 and September 2022. The extended timeline was due to COVID-19 restrictions, which prevented visitors from entering nursing homes. The author carried out all interviews in the participants’ rooms in the nursing homes, a setting chosen together with the older people to ensure both safety and privacy.

Before the interviews, the older people received written information about the study along with consent forms. The older people were informed that their participation was entirely voluntary, with the option to withdraw without repercussions. During a face-to-face meeting, the author provided additional explanations, and written informed consent was obtained from each participant.

To assess cognitive ability, the author first administered the Swedish version of the S-MMSE. Only older people scoring above 20, which is indicative of normal cognitive functioning, were included in the study (Palmqvist et al., 2011). A semi-structured interview guide was used to facilitate the conversations. Open-ended questions included prompts such as: “*How do you feel when you go to sleep*”, “*Can you describe your sleep problems*”, “*What sleep problems do you feel you have*”, and “*What do you think is causing your sleep problems*”. Follow-up questions, such as “*Can you tell me more*” and “*What do you mean by that*”, were used to encourage more profound responses. A pilot interview was conducted to refine the questions, which led to minor adjustments to two questions to ensure greater clarity. One of the original questions, “Do you have any sleep problems?”, was found to be too general and could lead to very short or yes/no answers. Another question, “Do you feel tired?”, was considered too direct and could give a simple “yes” or “no” answer. The pilot interview was included in the final dataset. Each interview lasted an average of 15 minutes (range 10–30 minutes) and was digitally recorded and transcribed verbatim to ensure accuracy.

### Data^1^1.Please find the interview guide attached as Appendix analysis

The interviews were analysed using qualitative content analysis based on Graneheim and Lundman’s (2004) approach. This method, which involves dividing text into “meaning units” and condensing them while retaining the original content, has been widely used in qualitative research (Graneheim & Lundman, 2004). The author personally transcribed all interviews verbatim and carefully read the transcripts several times to develop a comprehensive understanding of the material. To maintain the integrity of the participants’ voices, the author carefully analysed the transcripts, focusing on extracting and summarizing key categories.

Each meaning unit was assigned a code, resulting in the development of 20 codes through iterative comparisons. This process involved several rounds of refinement, during which the author systematically reviewed and revised the codes to ensure they accurately represented the data. Consistent self-reflection and adherence to the methodology ensured credibility, while systematically documenting the entire coding process strengthened reliability.

The final codes were grouped into subcategories and broader categories, illustrated in Table 2. This systematic approach ensured that the analysis accurately reflected the data while offering a clear framework for identifying key findings. Qualitative content analysis has been used in past studies to explore various categories and themes within healthcare, as well as to examine patient experiences. This demonstrates its relevance and applicability in qualitative research (Elo & Kyngäs, 2008; Telhede et al., 2024a; Telhede et al., 2022; Lundman & Graneheim, 2008).

**Table 2.** Example of the analysis process.

Meaning units	Condensed meaning units	Cod	Subcategories	Category
You must call the alarm […] I cannot bear to do anything. I feel a little left out. A good night is when I get to sleep in and don’t wake up too many times. But I didn’t last night. It depends greatly on how well I know the people who work at night. So I know how they act and stuff, depending on how they are I know if I can raise the alarm or not.	Anxiety about alarm response and unpleasant behaviour of night workers can lead to a feeling of being a burden. This disrupts sleep, where the quality of rest depends on familiarity with the night workers.	Feeling of being a burden	External disturbance	Disturbing influence on sleep

## Ethical considerations

This study adhered to the principles outlined in the World Medical Association Declaration of Helsinki (World Medical Association, 2013). Participants received both written and verbal information regarding the study’s purpose, emphasizing the voluntary nature of their participation and their right to withdraw at any time without providing a reason. Confidentiality of the data was ensured, and the study was approved by the Swedish Ethical Review Authority in Gothenburg, Sweden (Dnr. 2019–03817).

## Results

The results formed two categories: *Valuing good sleep* and *disturbing influence on sleep*, with the subcategories of *internal* and *external disturbances.*

### Valuing good sleep

The older people emphasized the vital role of good sleep, describing it as fundamental to their overall well-being. They shared that uninterrupted sleep was key to maintaining their health and providing the energy and strength necessary for daily activities. They expressed how their bodies seemed to naturally signal when it was time to rest, often feeling an increased sense of fatigue as a cue to sleep. They stated that sleep was essential for physical recovery, mental clarity, and emotional resilience.

I usually go to bed when I feel sleepy. But every day is different, depending on what the day has been like[…] Yes, I don’t really think much about it, sleep determines whether I’ll fall asleep or not[…] Of course, sleep is important. Without it, you’d be tired all day. You feel refreshed when you sleep well. (Woman, 87 years)

When sleep came quickly and undisturbed, they described it as a restorative experience, significantly enhancing their quality of life. However, when sleep problems arose these disturbances took on a profound weight, amplifying their need for rest and increasing the strain they felt in facing daily challenges. As sleep issues became more frequent, frustration grew, and many found themselves struggling to achieve enough rest, leaving them drained and vulnerable. One woman reflected on her struggle with sleep, saying.

Now it’s maybe two or three hours a night, and I am happy if I get to sleep even that much. (Woman, 77 years)

The older people emphasized the importance of a good night’s sleep, describing it as a cornerstone of physical and mental revitalization. They said that when they could sleep well, it became a vital source of restoration, providing a sense of vitality that permeated their entire being. A full night’s sleep allowed them to rest peacefully and wake up rejuvenated, ready to embrace the day ahead—something they greatly valued.

A full night’s sleep allowed me to rest peacefully and wake up rejuvenated, ready to embrace the day ahead—Sleep combines wakefulness, activity, and rest and sleep. And if you have a good relationship between those factors or pieces together […] So when you go to bed you sleep and when you wake up you wake up and you are alert. And it happens automatically. It is not something you switch on and off. It is usually the case that if you go to bed early, you can wake up a little early […] Then you get up and start doing something that is in front of you. (Man, 85 years)

They also shared that when they struggled with sleep at night, they often resorted to napping during the day to make up for lost rest. However, many feared going to bed, knowing they might face another restless night. This fear added to their anxiety and made the prospect of sleep feel even more daunting. They experienced this revitalization as closely linked to their ability to maintain daily routines and overall well-being, reinforcing the idea that good sleep is essential for a meaningful and functional life, especially in the context of ageing.

If you don’t do anything during the day, it’s boring and nothing happens. I’m almost 90, but it’s so important that you sleep properly to get through the day and be rested… If I sleep during the day, I’m happy with the sleep, but then I’m awake at night. (Woman, 89 years)

The older people stated that the importance of sleep became particularly clear when it was disrupted. During these times, the lack of rest felt like a loss of stability, leaving them physically exhausted and mentally foggy. They said that the quality of sleep was crucial, and when it was poor it significantly influenced their daily lives, resulting in constant fatigue. The participants stated that the more they struggled with sleep, the more they valued its importance, creating a negative cycle where their desire for rest grew stronger, but the frustration of not achieving it made sleep even more difficult to attain.

One respondent said: “*I am 101 years old, and after a few nights of not being able to sleep, I feel like I am 200 years old instead*” (Man, 101 years).

The older people experienced the emotional burden of insomnia, noting that it not only drained their energy, but also distorted their sense of age and vulnerability. As their sleep quality declined, their sense of powerlessness grew, making daily life even more challenging. They stressed that the lack of good sleep did more than just sap their physical energy—it eroded their independence, making them feel frail and reliant on others.

*“I feel a bit left out sometimes when I haven’t slept well. When I get to sleep in, I don’t feel that way[…] but waking up too many times makes me feel left out.” (*Woman, 85 years).

## Disturbing influences on sleep

### Internal disturbances

The older people talked about the strong connection they felt between their inner experience of ageing and the changes that occurred in their sleep patterns. They noticed that insomnia had become a familiar and almost expected part of ageing. They mentioned that they felt that they generally needed less sleep than younger individuals; however, they also said that as their physical health declined, their anxiety began to increase, and this worsened their sleep problems. They said that this inner disturbance, as they described it, became a vicious cycle: the more anxious they felt about not sleeping, the harder it became to relax and rest.

Yes, I guess it’s age-related. I’ve been through a lot, and now I have time to sit and think differently than before. I think I’m getting a little more anxious. I’ve started to wonder, “Will I be able to sleep tonight because of the anxiety?” And when I don’t sleep, the anxiety comes—something I never thought about before. (Woman, 91 years)

The older people said that heightened internal anxiety and bodily restlessness often made it difficult for them to relax and fall asleep. They described experiences of fragmented sleep that led to anxiety, resulting in long nights spent lying awake and staring at the ceiling. The older people said that internal nervousness frequently led to multiple awakenings throughout the night, even when they initially managed to fall asleep. They shared how they found themselves tossing and turning, unable to settle down, and instead of returning to bed they wandered around their rooms at night.

I wake up, and it’s this panic. It only comes when I wake up, and then I can’t stay in bed. I simply have to get up and walk around. After a while, I can sit in an armchair, but it’s a restless feeling that doesn’t let me settle. (Man, 67 years)

The older people said that this restlessness also often prompted them to get up and go to the bathroom, a behaviour they explained by believing that they needed to relieve themselves even when they did not. They said that these frequent bathroom trips were because of their anxiety and discomfort, interpreting them as necessary actions due to their inability to sleep. As one informant reflected,

If you have a restless soul, it spills over into sleep. (Man, 85 years)

The older people expressed that night-time restlessness often led to insufficient and fragmented sleep, leaving them feeling tired and unrefreshed. To cope with this, they said that they would take short naps during the day to compensate for their lack of rest. However, they also experienced tension between the need for daytime napping and their desire to avoid disrupting their night-time sleep. They explained that while napping provided temporary relief, it could interfere with their ability to fall asleep later, creating a cycle of disrupted sleep patterns. The older people said that sedative medications were occasionally suggested as a way to manage their night-time anxiety and restlessness. While they experienced the benefits of these medications, they often experienced them as unsatisfactory or inadequate solutions. They described feeling dizzy or even more tired after using sedatives, which they felt undermined their overall sense of well-being.

I used to take a sleeping pill and a sedative every night. But the other day, they told me I shouldn’t take the sleeping pill every night because it loses its effect. […] The fatigue can last into the next day too. I’ve been very careful to only take one every night when I go to bed. (Man 101 years).

They stated that physical discomfort, such as persistent pain, contributed to their sleep difficulties. They said that lying in bed for extended periods often exacerbated these problems, making it harder for them to relax and fall asleep. Their frustration was similar as they described the challenges of balancing their need for rest with the discomfort they experienced at night. One woman explained,

You feel better if you sleep better; five or six hours, yes, five hours. However, my back hurts; I lie down for a long time before I fall asleep, and I hardly sleep at all. (Woman, 91 years)

The older people expressed how negative thoughts and reflections often disrupted their ability to fall asleep, particularly during the quiet and solitary night hours. They said that these thoughts frequently centred on existential concerns, such as fears of death, regrets about the past, and an acute awareness of their diminishing time. They explained that the stillness of the night often magnified these worries, leaving them restless and unable to calm their minds. They said that these mental preoccupations created a significant barrier to falling asleep, and they described feeling isolated in their struggles because these reflections were difficult to express to others.

Yes, of course, it’s the thoughts that keep me awake […]. They wander back and forth because my situation is different now than it used to be. Life is shrinking. Time is shrinking in every way—everything is slowing down. (Woman, 92 years)

They said that nightmares were a recurring source of distress, waking them abruptly and leaving them anxious and unsettled. They explained that these vivid and often unsettling dreams sometimes stemmed from unresolved fears or past traumas, making it difficult to relax enough to return to sleep. They said that the anticipation of experiencing nightmares created a sense of dread about going to bed, further complicating their ability to achieve restful sleep. One woman summarized these challenges, reflecting on how her thoughts kept her awake:

Falling asleep is the worst. It is hard to fall asleep […] Why is it the way it is, life and what has been? I have had a bit of a hard time because these thoughts can come at any time, but especially at night. (Woman, 77 years).

## External disturbances

The participants experienced external changes in life, such as retirement, that mark significant life transitions. They said that the departure from their long-standing roles and daily routines often left a void, contributing to decreased activity levels. This decline was, in turn, perceived as an aspect that accelerated the onset of sleep problems, such as difficulty falling asleep or staying asleep through the night. They said that retirement reduced their physical activity and disrupted the structured rhythm of their days, making it more challenging to maintain regular sleep patterns. They said that engaging in activities during the daytime was crucial to achieving quality sleep at night. Engaging in purposeful activities during the day helped them feel physically tired and mentally satisfied at night.

It’s hardest to fall asleep at night. When I was young, I could sleep straight away—just put my head on the pillow, and it was good night. […] I was a maid, then I worked at[…] what’s it called? A nursery. Now I just lie there thinking—back then, so much happened. (Woman, 87 years old)

They said that enjoyable activities such as listening to music or participating in light physical exercises provided by the nursing home gave them a feeling of relaxation, which facilitated the possibility of better sleep. The older people experienced how their opportunities for meaningful activities gradually decreased as they aged. They said that this decline had a negative influence on them and negatively affected their sleep. They expressed how reduced mobility or health concerns limited their ability to participate in activities they once enjoyed. They also expressed that the lack of diverse and stimulating options in the nursing home made them feel unmotivated, which they experienced as further exacerbating their sleep difficulties. One woman reflected,

I used to be out and about so much before. I was so tan and tired naturally […] That’s what you get when you work a lot. (Woman, 98 years)

Furthermore, the older people said that external factors, such as the nature of their social lives, considerably influenced their sleep conditions. They said that well-functioning and supportive relationships with family and friends encouraged an environment conducive to restful sleep. However, they said that their loneliness often increased when moving into nursing homes, particularly following the loss of loved ones, which contributed to insomnia. An example came from a woman who expressed,

Something worries me when I don’t sleep, but I don’t really know what it is. There have been a few things. First, people you knew have passed away, then the worries get worse. (Woman, 91 years)

The older people said their sleep was generally better in familiar environments, such as their homes, compared to nursing homes. They experienced that having self-control over their bedtime and evening routines greatly improved their sleep quality. They said that a sense of security with the nursing staff also emerged as a crucial factor, and they said that having negative feelings towards the nursing staff directly influenced their ability to relax and sleep. Some described feeling uncertain and uncomfortable with specific nursing staff, particularly those who appeared stressed or who approached them loudly. They said they often knew which nursing staff were on duty at night, and this influenced their willingness to seek assistance. They said that they avoided calling for help when specific nursing staff were working, which contributed to a heightened sense of insecurity and fear during the night, further disrupting their sleep and overall well-being. As one older man explained,

It has a lot to do with how I feel about the night workers, knowing how they behave. Then I know if I can raise the alarm. (Man, 67 years)

Additionally, they experienced external disturbances at night, such as noise from other residents or nursing staff entering and exiting their rooms, which disrupted their sleep. They said that bright lights and disturbing sounds exacerbated their inability to sleep and made them insecure about disturbing the nursing staff during the night, deterring them from asking for help even when needed.

## Discussion

The older people in this study reported feeling that a good night’s sleep is essential to maintaining energy and coping with daily activities and is crucial to their overall health and well-being. However, their experiences of insomnia, characterized by difficulty falling asleep, staying asleep, or waking up too early, negatively affected both their mental and physical health. These findings are consistent with previous research that links improved sleep to better health outcomes and quality of life among older people (Hirshkowitz et al., 2015; Kobayashi et al., 2021). The results of this study highlighted the circular relationship between anxiety and insomnia disturbances among older people in nursing homes. Internal disturbances, including emotional and physical factors, combined with normal ageing, led to an increase in feelings of anxiety and restlessness, which in turn exacerbated insomnia or vice versa. The older people in this study described how anxiety created a vicious cycle, where the inability to sleep increased their anxiety, and their anxiety further disrupted their sleep. These findings support existing research indicating that anxiety and related health problems significantly affect insomnia, particularly in older populations (Krahn et al., 2020). Moreover, other studies have found that insomnia in older adults is strongly linked to elevated levels of anxiety, highlighting the bidirectional nature of this relationship (LeBlanc et al., 2020).

Additionally, this study showed that older people who were unable to sleep expressed significant concern about their inability to sleep, which suggests that their experience of inadequate sleep led to heightened anxiety. The sleep problems at night was sometimes accompanied by compensatory daytime rest. These findings align with research suggesting that individuals experiencing insomnia frequently engage in maladaptive behaviours, such as oversleeping, that further disrupt their sleep-wake cycle. This phenomenon has been observed especially in older people, where anxiety about sleep leads to both poor quality night-time sleep and excessive daytime sleep, thus compounding the issue (Roth et al., 2021).

The results also demonstrated how chronic health conditions, which are common among older people in nursing homes, contributed to insomnia. Chronic illnesses not only directly affect sleep, but also exacerbate feelings of anxiety, making it even more difficult to get good sleep. Previous research by Patel et al. (2018) emphasized the detrimental consequences of chronic health conditions on sleep, with conditions such as arthritis, heart disease, and respiratory problems often leading to insomnia. More recent studies have also confirmed that chronic illnesses like diabetes and cardiovascular disease significantly contribute to insomnia in older adults (Wright et al., 2023). These findings indicate that the relationship between insomnia and anxiety is complex and multifaceted, with each factor reinforcing the other, thereby creating a vicious cycle (Smith & Williams, 2022).

External disturbances play a substantial role in shaping sleep experiences among older people in nursing homes. Older people experienced things that reduce activity levels, such as retirement, loneliness, and unfamiliarity with the nursing home environment, all of which contributed to insomnia. This is consistent with a previous study showing that retirement often leads to reduced engagement in meaningful activities thus negatively influencing sleep (Zhang et al., 2022). In addition, the participants described the importance of familiar routines and supportive social relationships in promoting better sleep. This key finding mirrors previous studies emphasizing the influence that social connectedness and routines have on sleep quality (Gupta et al., 2022; Hirshkowitz et al., 2015).

The physical environment of the nursing home also plays a decisive role in the quality of sleep. Older people in this study experienced that noise, light exposure, and nocturnal disturbances from nursing staff interfered with their ability to sleep restfully. These findings are consistent with previous studies, where environmental disturbances are commonly reported as barriers to good sleep in nursing homes (Gupta et al., 2022; Li et al., 2021).

This study showed that older people often feel insecure around some nursing staff, which hinders their sleep and their ability to relax. In previous studies, empathetic and responsive nursing staff behaviour was shown to significantly reduce patient stress. Trust and safety in nursing staff are crucial elements influencing older people’s ability to relax and achieve restorative sleep, and negative experiences or perceptions of nursing staff behaviour can create obstacles to relaxation and restfulness. Also, a study by Cap et al. 2024)emphasized that trust in nursing staff promotes a safe nursing home environment, which makes patients feel more at ease and makes them better able to manage their health conditions. Conversely, feelings of uncertainty or mistrust can lead to increased anxiety, which is detrimental to insomnia. Similarly, research by Hoyer et al. (2021) indicates that patient satisfaction and emotional well-being are directly linked to their trust in and comfort with nursing staff, especially during vulnerable times such as night-time care. The older people in this study genuinely preferred quiet, familiar surroundings with limited interruptions, thus reinforcing the need for person-centred interventions to address the internal and external factors that affect insomnia (Blytt et al., 2020). This study highlights the complexity of insomnia in nursing homes, emphasizing factors such as noise levels, light exposure, unfamiliar surroundings, internal anxiety, the effects of ageing, and the relationships between nursing staff and residents. According to Huang et al. (2020) the prevalence of insomnia varies across nursing homes due to differences in staff education, resident needs, and environmental conditions. Research by Yang et al. (2022) suggests that optimizing factors like reducing night-time noise, adjusting lighting to support circadian rhythms, and creating familiar environments can improve sleep quality.

Furthermore, structured training for nursing staff in sleep-promoting care strategies can increase knowledge and improve insomnia management (Dew et al., 2020). Nonpharmacological interventions, such as cognitive behavioural therapy for insomnia (CBT-I) and relaxation techniques, have been shown to effectively reduce insomnia symptoms (Yeung et al., 2018). The heterogeneity of nursing homes—ranging from staff training to care routines and physical environments—plays a significant role in the prevalence of insomnia.

Future research should explore how facility size and the alignment between nursing staff knowledge and resident personalities affect insomnia, as this could lead to more targeted interventions and a deeper understanding of sleep disturbances in such environments.

## Strengths and limitations

In this study, the researcher was solely responsible for all stages of the research process, including data collection, analysis, and interpretation. While this ensured a consistent and unified approach, it also introduced a potential limitation due to the absence of collaborative perspectives that might have enriched the analysis. The lack of triangulation with other researchers meant the findings relied heavily on the researcher’s interpretations, increasing the risk of bias. However, the author engaged in continuous reflexivity throughout the process, critically reflecting on their pre-understanding to minimize its influence on the results (Malterud, 2001). Acknowledging the researcher’s background, particularly in nursing home conditions, can help clarify how this influenced the study. This reflexive approach strengthens the study’s transparency and supports confirmability (Polit & Beck, 2018). However, the lack of external collaboration may have restricted the depth of the analysis. While this prior knowledge enabled a nuanced approach to data analysis, it also necessitated vigilance to avoid unintentional bias. To enhance confirmability, the author adopted an inductive approach to qualitative content analysis and incorporated direct quotes from participants, ensuring that findings were firmly grounded in their experiences (Elo et al., 2014; Polit & Beck, 2018).

The author designed and conducted the semi-structured interview format, providing a structured yet flexible framework for data collection. A pilot test of the interview guide refined the questions, improving their clarity and enhancing the depth of responses. Data saturation was achieved when no new information emerged, and additional interviews were conducted to confirm data completeness, further strengthening the findings’ credibility (Polit & Beck, 2018).

A limitation of this study was the lack of clarification regarding participants’ cognitive abilities, which affected the transparency of the data collection process. Specifically, knowing how many participants self-reported symptoms of mild cognitive impairment or had a formal dementia diagnosis could have strengthened the credibility of the findings, as cognitive status may influence the ability to recall sleep experiences. However, a strength of the study is its ability to capture participants’ lived experiences of sleep, regardless of cognitive status, providing valuable insights into their subjective perceptions. The use of the MMSE as a screening tool was appropriate. However, a more detailed assessment of cognitive status would have enhanced the transferability of the results by offering greater contextual depth (Malterud, 2001)

Nineteen interviews across seven nursing homes, conducted in participants’ private rooms, added credibility by creating a familiar and comfortable environment for data collection (Malterud, 2011; Polit & Beck, 2018). However, the sample’s gender imbalance, with fewer male participants, posed a limitation. While this reflects typical demographics in nursing homes, a more balanced gender representation might have offered additional insights. Additionally, it would be beneficial to discuss how potential cognitive impairments among participants might have affected their ability to provide accurate and consistent responses, which could influence the dependability of the findings (Malterud, 2001).

Due to the study’s context-specific nature, the findings primarily contribute to contextual validity and may be most applicable to similar nursing home settings (Malterud, 2001). While patterns across interviews indicate transferability to other nursing homes, the dependability of the results beyond this context remains uncertain (Polit & Beck, 2018). Future research could explore the influence of cognitive impairments on patient experiences to strengthen the transferability of the findings. Additionally, studying other elements, such as length of stay, activity levels, or sleep medication use, could enhance perspective triangulation by providing deeper insights into how sleep experiences are shaped in nursing homes (Malterud, 2001).

This study did not gather specific data on factors such as participants’ length of stay in the nursing home, activity levels, or the use of sleep medications. These variables could influence sleep experiences and, therefore, warrant further exploration. For instance, the length of stay may influence older people’s adjustment to the care environment, while activity levels and sleep medication use could directly influence sleep quality. According to Polit and Beck (2018), reflexivity is crucial in qualitative research, and recognizing these limitations strengthens the study’s transparency. Including these factors in future research could provide additional insights into how sleep experiences are shaped in long-term care settings, thereby enhancing the transferability of findings (Graneheim & Lundman, 2004). Subjective and objective sleep data, including demographic variables like age, could have further contextualized the results. Data triangulation could improve the credibility and dependability of future findings (Malterud, 2001). The broad age range of participants may have influenced sleep experiences, particularly in older people, where changes in sleep patterns are well-documented (Hirshkowitz et al., 2015). Additionally, future studies could consider the role of cognitive impairment, as the ability to recall and report sleep experiences might vary significantly among participants with dementia or mild cognitive impairment.

## Conclusion

This study highlights the importance of sleep for older people in nursing homes, emphasizing its role in maintaining energy, coping with daily life, and enhancing overall well-being. However, both internal factors, such as anxiety and chronic health problems, and external factors, including environmental disturbances, uncertainty about caregivers, and inadequate support from caregivers, disrupts their sleep. These findings underscore the critical need for care in which older people feel safe and supported. Trust and positive interactions with caregivers are crucial for reducing anxiety and promoting a supportive sleep environment. By addressing these internal and external barriers, nursing homes can implement tailored interventions to improve sleep quality, ultimately improving the health and quality of life of older people.

## Data Availability

The data underlying this study’s results are securely stored at Halmstad University. Although these datasets are not publicly accessible due to institutional limitations, they can be made available upon request. Requests for access should be directed to the corresponding author.

## Appendix

### AppendixInterview guide

“Can you tell me a bit about your daily routine and your sleeping habits?”

“How would you describe a typical night’s sleep for you?”

“How do you feel when you go to sleep?”

“Can you describe your sleep problems?”

“What sleep problems do you feel you have?”

“What do you think is causing your sleep problems?”.“Do you find it hard to fall asleep, stay asleep, or wake up too early?”

“Are there any particular times of the night when you feel more awake or restless?”

“How do your sleep problems affect your daily life or activities?”

“Have your sleep problems affected your mood or energy levels during the day?”

“What do you think is causing your sleep problems?”

“Have you noticed any particular factors (e.g., environment, stress, health issues) that worsen your sleep problems?”

“Do you think age or other changes in your life have affected your sleep?”

“Have you tried anything to help you sleep better?”

“What have you found helpful in improving your sleep, if anything?”

“Do you feel like the nursing staff in the nursing home is supportive in addressing your sleep problems?”

### Follow-up questions

“Can you tell me more about that?”

“What do you mean by that?”

“How does that make you feel?”

“Could you explain that in more detail?”
